# Shaping Ability of Reciproc Blue Versus One Curve in Curved Canal: An In-Vitro Study

**DOI:** 10.7759/cureus.24387

**Published:** 2022-04-22

**Authors:** Mohammad Daher Altufayli, Basem Salim, Imad Katbeh, Razan Merei, Zarina Mamasaidova

**Affiliations:** 1 Department of Operative and Endodontics, Faculty of Dentistry, Tishreen University, Latakia, SYR; 2 Department of Pediatric Dentistry and Orthodontics, Peoples' Friendship University of Russia (RUDN University), Moscow, RUS

**Keywords:** one curve, reciproc blue, rotary motion, reciprocation motion, nickel-titanium, working length

## Abstract

The introduction of nickel-titanium (NiTi) rotary instruments improved the root canal preparation of the narrow and curved root canals, especially after the introduction of thermomechanical treated (NiTi) alloys that have a high flexibility to prepare the curved canal reducing the common clinical complication, such as fracture, the change of the original shape of the root canal as a result of the change in the curvature of the curved root canal, the change of the working length which caused by the creation of the ledges, canal transporting and zipping especially in medium and highly curved canal. This study aimed to compare the shaping ability of two heat-treated nickel-titanium single file systems using reciprocation motion and rotary motion in curved canals.

Materials and methods

Thirty extracted human teeth with one curved root at 25 to 56 degrees were used, two NiTi single file systems were used to prepare the curved root canal in two groups: Reciproc R25 (Munich, Germany: VDW) group (n=15) and One Curve (Besancon, France: Micro-Mega) group (n=15). Curved root canal instrumentation outcomes were evaluated using cone-beam computed tomography (CBCT). Kruskal-Wallis with Bonferroni post hoc test was used to assess differences between working length, the angle and radius of curvature after instrumentation.

Results

There was a significant difference in angle and radius of curvature (-1.83° One Curve, -2.25° Reciproc blue and -0.18 mm One Curve, -0.19 mm Reciproc blue, respectively) (p<0.05) after instrumentation for both One Curve and Reciproc blue groups, and there was no significant difference in working length change (-0.16 mm One Curve, -0.32 mm Reciproc blue) after instrumentation of both One Curve and Reciproc blue groups (p>0.05).

Conclusion

The Reciproc blue single file system with reciprocation movement and One Curve with continuous movement cause a significant difference in curvature and radius of curved root canal affecting the original shape of the root canal with no significant difference in working length of the curved root canal.

## Introduction

The aim of the mechanical preparation was to create a tapered space along the root canal for delivery of irrigation medication and finally, a three-dimensional seal of the prepared root canal system, the curved root canal anatomy (degree and radius of curvature) affects the preparation of curved root canal [1,2].

The preparation of curved root canal using nickel-titanium (NiTi) rotary instruments maintains the original shape of the root canal and provides a better clinical outcome than the preparation using stainless steel [3]. The introduction of NiTi alloys with thermomechanical treatment provides improved clinical performance of the curved root canal [4].

In the last few years, a new motion was introduced as an effective and safe motion to prepare the curved root canal which is reciprocation motion [5,6]. The reciprocation mode of Reciproc blue (Munich, Germany: VDW) cut in a counterclockwise motion, while the other root canal rotary instruments One Curve (Besancon, France: Micro-Mega) cut in a clockwise motion.

The reciprocal mode of preparation reduces the instrument binding in the root canal and increases the lifetime of the instruments compared to full clockwise rotating instruments [7,8]. The reciprocation motion increases the life span of the preparation instruments by reducing the stress values better than continuous rotation [5,9]. Recent studies showed that the reciprocation instrument makes an excellent result in preparation of the curved root canal [5,10,11] and reduction of the microorganisms in the root canal system [12,13].

VDW has recently modified the M-wire of the Reciproc to Reciproc blue with an innovative heat treatment that gives the instrument a blue color. This operation increased the flexibility, the resistance of cyclic fatigue, and caused less surface microhardness marks than the traditional M-wire [14]. This single file system with R phase has three main tip sizes (R25-R40-R50) with a taper of 08 starting after three millimeters from the end of the instrument. The Reciproc blue instruments have an S-shaped cross-section the instruments are used with a motor at 10 full cycles of reciprocation motion per second. The motor rotates with different angles of reciprocation and speed, the instrument cutting direction is forward rotation [15].

One Curve is a single rotary file system that is used for shaping the full length of root canal with a single instrument, this system is manufactured from heat-treated NiTi alloy. The new alloy is called C-wire and has the controlled memory advantage, pre-bendable, variable cross-section with continuous rotation motion. This single file system has a single design with a 06 taper and 25 tip sizes. Moreover, the manufacturer of the file states that C-wire technology lessens the instrument’s threading, sticking, and binding to the curved root canal walls which makes the root canal preparation easier [16].

A right working length determination and saving the original shape of the curved root canal during the endodontic treatment and root canal instrumentation are very important. It makes the clinical outcomes of endodontic treatment more successful [1].

There are many methods that have been used to evaluate the canal anatomy of the root canals, but there were always many difficulties in associating the curved root canals, until the use of the three-dimensional digital radiography cone-beam computed tomography (CBCT). Cone-beam computed tomography has been introduced especially for use in the facial areas, which has led to its widespread use in dental practice [17]. Recent studies have shown that most of the current research in the dental field use CBCT [17]. The CBCT image enables us to perform tests at a level ranging between 0.4 and 0.076 mm, due to the properties it provides on all sagittal, axial, and lateral planes, in addition to multiple levels that can be studied.

## Materials and methods

The study was registered by Tishreen University Council, Syria (protocol code: 559, date: November 2019). Sample size was calculated using outcomes from Staffoli et al. and Yufei et al., comparing many different single file systems with different motions [18,19]. Sample size calculation produced a required sample size of 30 curved roots to detect a significant difference (90% power, two-sided 5% significance level) followed by independent sample t-test at 5% significant level.

A total of 30 extracted human teeth with one curved root at 25-56 degrees were used, the curved root was separated with diamond burs from the coronal part at length of 12 mm for all canals and the canals were measured to the apical foramen with a size 10 C-file (Shanghai, China: Fanta Dental Materials Co., Ltd).

**Figure 1:**
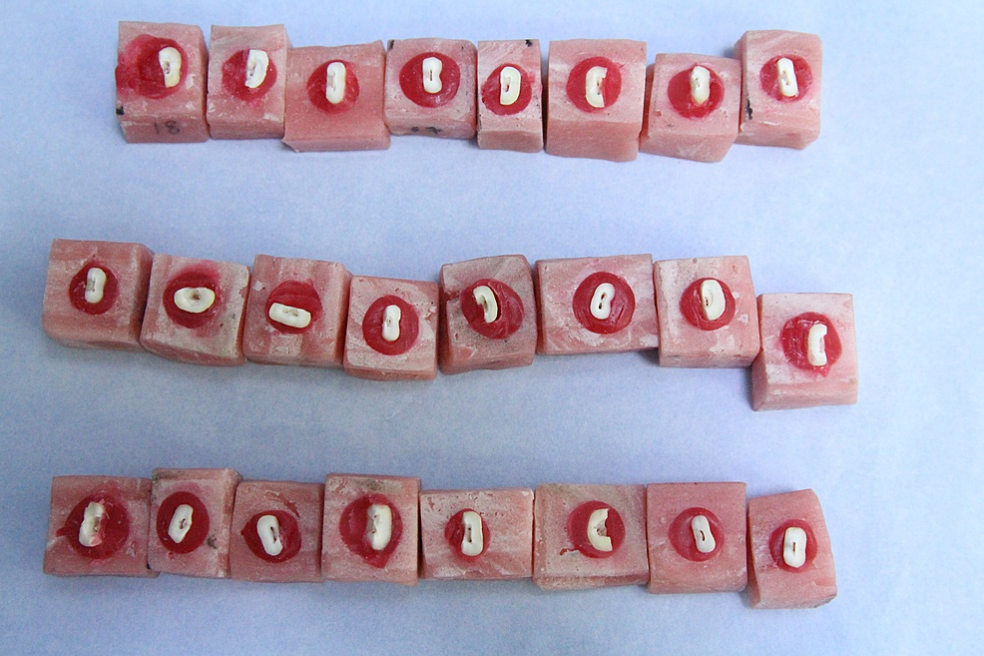
Implanting the teeth within the mold.

Only teeth with an intact root canal to the apical foramen without classification, previous treatment, and resorption were chosen. Each root canal of the samples was placed in a special cube-shaped mold made of polymethyl methacrylate. In order to maintain a stable position of the teeth during radiography and instrumentation, the teeth were put inside the mold and fixed with red wax (Figure 1).

Root canal instrumentation

The working length was 12 mm and a manual glide path up to ISO-size 15 was prepared using C-files. After each instrument, the root canal was irrigated with 3 mL of 5.25% NaOCl solution and at the end of instrumentation with 5 mL of NaOCl using a plastic syringe with 30 g close-ended needle. The needle was then inserted at 10 mm into the root canal without binding using peaking motion three times per second. Two NiTi single file systems were used to prepare the curved root canal in two groups.

Group of Reciproc R25 (N=15)

The selected size from the single file system was #25 tip size, 0.08 taper, and 25 mm length supplied by VDW, with a speed of 300 rpm, 30° clockwise, and 150° counterclockwise rotation angles, using automatic motor (Changzhou, China: Changzhou Sifary Medical Technology Co., Ltd.). This was done with 17% ethylenediaminetetraacetic (EDTA) and using peaking motion every 3 sec until the working length was reached.

Group of One Curve (N=15)

The only size from the single file system is #25 tip size, 0.06 taper, and 25 mm length supplied from Micro-Mega using rotary motion with speed 300 rpm, and a torque of 2.5 Ncm using automatic motor. This was done with 17% EDTA and continues motion until the working length was reached.

Determination of changes in working length and curvature angle

**Figure 2:**
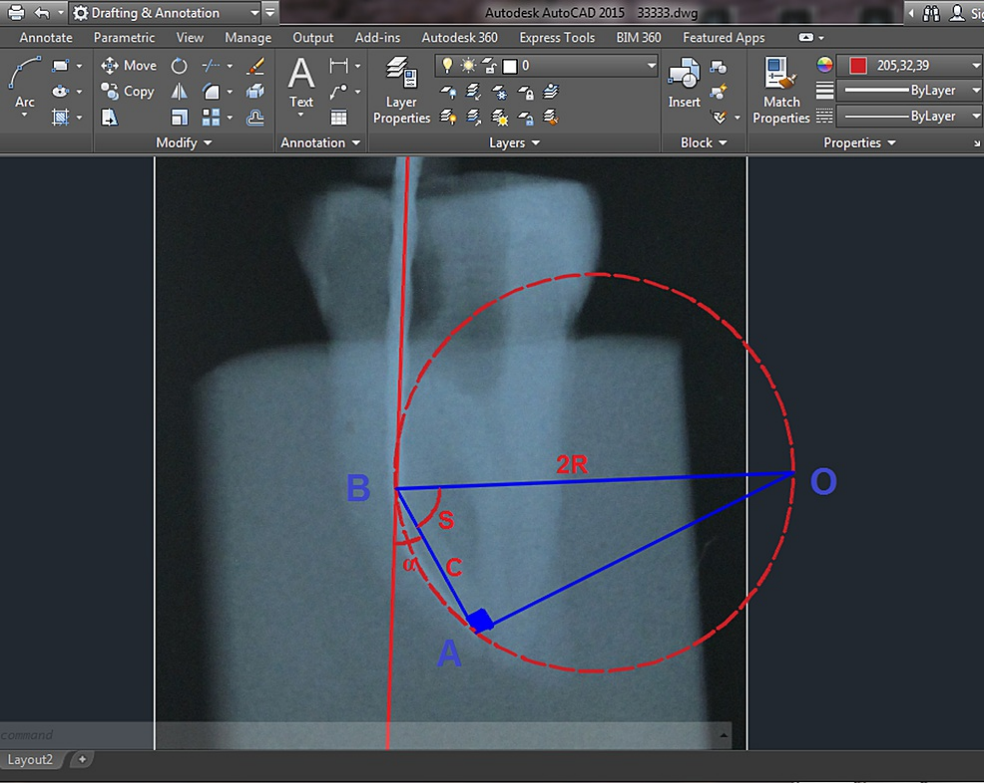
Illustrative image of the process of determining the radius of curvature.

CBCT images of the sample before and after preparation were taken, this method proved highly effective in evaluating morphological changes to the original shape of the root canals, especially the curved root canals, the CBCT program (BlueSkyPlan software; Libertyville, IL: Blue Sky Bio) provides many tools that allow accurate measurements for working length and curvature angle [11]. The program provided the possibility to determine the working length and curvature angle using three-mark points (www.blueskyplan.com). The first point was the starting point of the coronal root (D), the second point was the curvature point (B), and the third point was the end of the curved root canal (A). The radius of curvature was calculated using the equation: R = C/(2 cosine S). Here, C was the straight line between the A and B, the S angle equals (90 - a), and a was the curvature angle (Figure 2).

Evaluation

**Figure 3:**
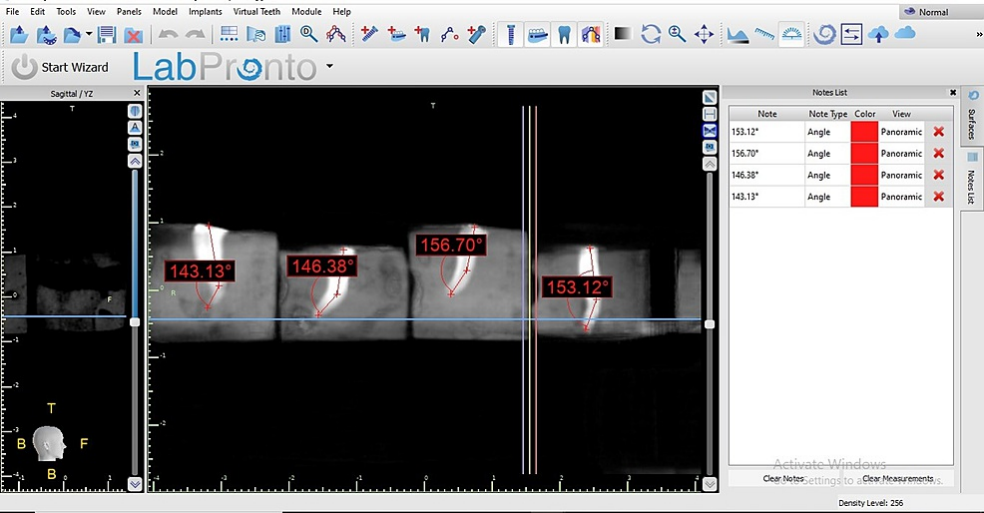
The curvature angle determination before instrumentation.

**Figure 4:**
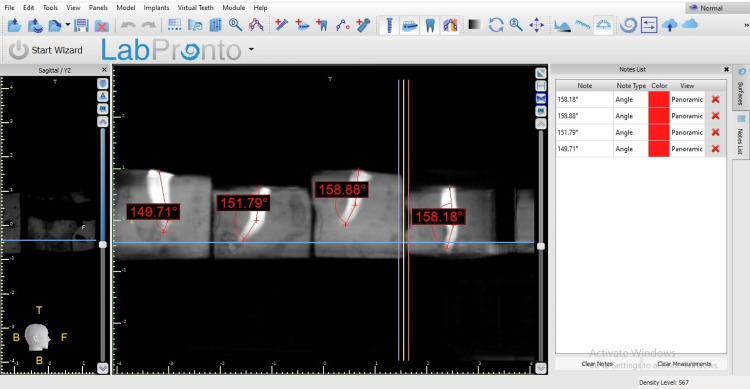
The curvature angle determination after instrumentation.

**Figure 5:**
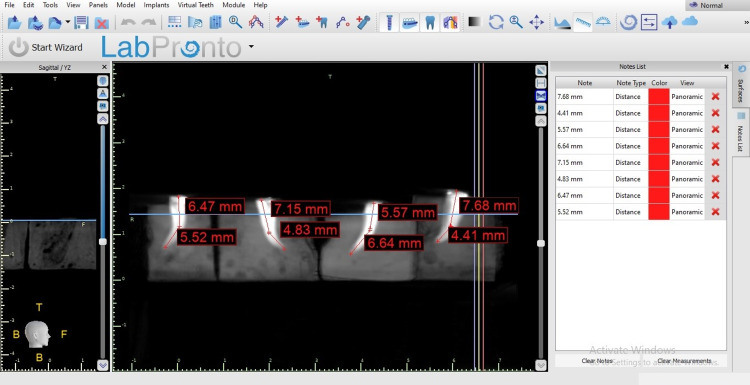
The working length determination before instrumentation.

**Figure 6:**
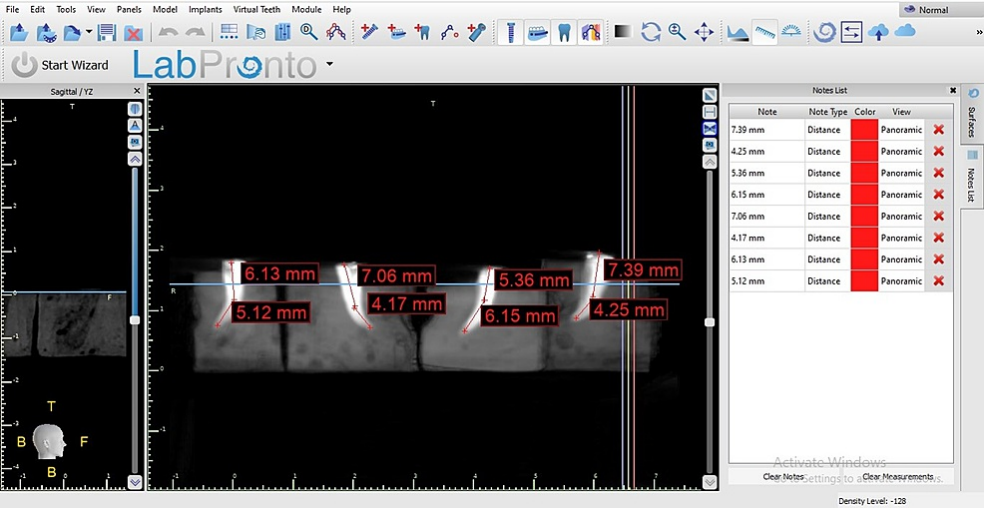
The working length determination after instrumentation.

All curved root canal instrumentation was completed by one examiner and evaluated the outcomes using CBCT by second examiner to avoid personal bias. VATECH CBCT machine (Gyeonggi, South Korea: VATECH Co., Ltd.) was used to evaluate the outcomes of the canal instrumentation (Figures 3-6).

Statistical analysis

Kruskal-Wallis with Bonferroni post hoc test was used to assess differences between working length, the angle and radius of curvature after instrumentation. The statistical program SPSS version 20.0 (Chicago, IL: IBM Corp.) was used for data analysis. The results were considered significant if p≤0.05. Sample size was determined using a sample size calculation program (PS Power and Sample Size Calculation Program, Version 3.0.43 {William DD and Walton DP Jr. - Vanderbilt University: Nashville, TN}).

## Results

The curvatures of all root canals ranged between 25° and 56° (Table 1), and the radius ranged between 0.98 mm and 2.90 mm (Table 2). During instrumentation of the curved root canals, no instrument fracture or deformation was noted. None of the prepared root canals was blocked with debris.

**Table 1:** The mean values of canal curvature (degrees). *P-value <0.05.

The study groups		No. of the study group	Mean	Minimum value	Maximum value	Standard deviation	p-Value
One Curve	Before preparation	15	36.25	28.00	48.00	6.43	-
	After preparation	15	34.42	26.00	45.00	6.01	0.002*
Reciproc blue	Before preparation	15	38.42	26.00	56.00	9.86	-
	After preparation	15	36.17	25.00	53.00	9.29	0.002*

**Table 2:** The mean values of canal curvature radius (mm). *P-value <0.05.

The study groups		No. of the study group	Mean	Minimum value	Maximum value	Standard deviation	p-Value
One Curve	Before preparation	15	1.76	1.21	2.59	0.41	-
	After preparation	15	1.57	1.08	1.94	0.31	0.002*
Reciproc blue	Before preparation	15	1.76	0.98	2.90	0.54	-
	After preparation	15	1.58	0.93	2.60	0.48	0.002*

There was a significant difference in angle and radius of curvature (p<0.05) after instrumentation for both One Curve and Reciproc blue groups (Tables 1, 2) and there was no significant difference in working length change after instrumentation of both One Curve and Reciproc blue groups (p>0.05) (Table 3). Reciproc blue group showed better saving of the original shape of the curved root canal, which was presented in this study as the difference in angle and radius of curvature.

**Table 3:** The mean values of working length change (mm). *P-value >0.05.

The study groups	No. of the study group	Mean	Minimum value	Maximum value	Standard deviation	p-Value
One Curve	15	0.16	0.00	0.60	0.24	0.308*
Reciproc blue	15	0.32	0.00	0.60	023	0.308*

## Discussion

Extracted human teeth were used, because of the limitation of using the resin-made teeth due to the difference in hardness between dentin and resin [20]. In general, most of the studies that aim to evaluate root canal preparation, especially rotary preparation depended on extracted human teeth. More than 93.2% of the studies used extracted human teeth and 6.8% of them used resin casts, despite the wide anatomical differences when implementing extracted teeth, such as the nature and hardness of the dentin, the degree of curvature, and the classification amount [21]. However, the resin channels allow for standardizing all the variables such as the degree, location, radius of curvature, hardness, and size of the canals used in the study [21].

It has been suggested that evaluation of the changes on canal shape elements such as curvature and working length after instrumentation is a reliable process to assess the ability of rotary system to preserve the original shape of curved root canal [22].

In this study, the CBCT was used to evaluate the shaping ability of the tested groups. The CBCT imaging method is a safe and reliable process for the study of canal anatomy and for the analysis of shaping techniques before and after instrumentation [23,24].

Changes in canal curvature (angle and radius) after the use of the two different NiTi single file systems were statistically significant. This agrees with the findings of previous studies which found that both motions (continuous rotation, reciprocating motion) make a significant difference in the original shape of the curved root canal [22,25]. The reason for these changes may be the high range of curvature of the selected root canals, which are often narrow canals, and the large taper sizes of both single file Reciproc blue and One Curve. The variable cross-section of One Curve and continuous motion direct to the apical foramen may have caused more changes in the root canal curvature than the Reciproc blue which implements reciprocation motion and have an S-shape cross-section. Even though, when using the 0.08 taper Reciproc blue file it caused less influence because of the short working length we studied.

Other studies found that the rotary motion was safer and better in saving the original shape of the root canal than the systems with reciprocation motion because of the high effectiveness of dentin removal in the reciprocation motion systems [26,27]. The study by Nazari et al. in 2014 and the study by Gergi et al. in 2014 showed that power-driven preparation systems that depend on both reciprocation movement and continuous movement are able to maintain the original shape of the canal. These studies depended on studying the change in the curved canals of the mesial roots of the lower molars by micro-CT technique [28,29].

In another case, Sharma et al. in 2016 adopted micro-computed tomography to assess the shape of the canal after preparation, as it showed that systems that depend on both movements in preparation, reciprocation movement, and continuous rotational movement, led to a change in shape. The reason for this difference may be due to the study of a larger number than the number used in the canals, where 180 curved canals were used, which is almost five times the sample we study, and also to the use of micro-CT in the evaluation [30].

The study by Berutti et al. in 2012 showed that the preparation systems with reciprocation movement are better than those with continuous rotational movement in terms of preserving the original shape of the curved root canal, as the preparation systems with continuous rotational movement led to a fundamental change in the original shape of the curved canal, which may be due to the difference in the alloy of preparation systems between the thermally modified nickel-titanium used in the reciprocation motion system and the traditional nickel-titanium in the rotary motion system [31].

The change in the working length occurs during the preparation of the root canals in general, and the rate of its occurrence increases in the curved root canals, especially those with medium and high degrees of curvature greater than 15 degrees. This is because of the increased complexity in the morphological anatomy of the curved canals, which affects the ability of the rotary files to reach the entire working length towards the apical foramen, and also on its ability to perform adequate and appropriate cleaning and preparation of the root canal. The change in the working length often occurs because of failure to make an appropriate straight root canal access, the incorrect assessment of the curvature before clinical procedure, presence of canal calcifications and necrotic pulp remains, and failure to perform adequate and effective irrigation during preparation operations [32].

In this study, there was no significant difference in working length after instrumentation of both One Curve and Reciproc blue groups. This is consistent with most of the studies that studied the change in the working length when preparing curved root canals, whether in systems that depend on reciprocation movement or continuous movement, which included a second premolar and lower first molars, similar to the sample studied in our study [21,33,34].

Based on the limitations of the study we emphasize the importance of the future study of the clinical application of these systems to curved canals; the importance of comparing the time spent for both the reciprocation movement and the continuous movement to complete the preparation; the ability to access the posterior teeth, especially on patients with a small mouth opening cavity; and the evaluation of the preparation during the stages of canal filling, which indicate the effectiveness of the preparation.

## Conclusions

Under the circumstances of this study, the Reciproc blue R25 -0.08 single file system with reciprocation movement and One Curve #25-0.06 with continuous movement cause a significant difference in curvature and radius of curved root canal affecting the original shape of the root canal with no significant difference in working length of the curved root canal.
